# Mental disorders and psychological problems of women-victims of domestic violence during self-isolation in the covid 19 pandemic

**DOI:** 10.1192/j.eurpsy.2021.1601

**Published:** 2021-08-13

**Authors:** M. Kachaeva, N. Kharitonova, S. Shport, O. Shishkina

**Affiliations:** 1 Forensic Psychiatry, Federal State Budgetary Institution «V. Serbsky Federal Medical Research Centre for Psychiatry and Narcology» of the Ministry of Health of the Russian Federation, Moscow, Russian Federation; 2 Psychiatry, Moscow Research Institute of Psychiatry – a branch of V.Serbsky National Medical Research Centre for Psychiatry and Narcology, Moscow, Russia, Moscow, Russian Federation; 3 Psychiatry, Federal State Budgetary Institution «V. Serbsky Federal Medical Research Centre for Psychiatry and Narcology» of the Ministry of Health of the Russian Federation, Moscow, Russian Federation

**Keywords:** domestic violence victim, women, mental health, covid 19 pandemic

## Abstract

**Introduction:**

Domestic violence is a troubling problem, but it has acquired a new, previously unknown significance during the COVID 19 pandemic. According to WHO, the number of calls from victims of domestic violence to hotlines during quarantine in many countries has increased by an average of 5 times.

**Objectives:**

The purpose of this study was to find out the consequences of domestic violence against women and to identify psychological problems and mental disorders. Attention was paid to the mental health of victims of violence in self - isolation during COVID-19 pandemic.

**Methods:**

We analyzed the psychological problems and mental disorders of women who applied to the newly created hotline for women at the Serbsky Center in Moscow during the COVID 19 pandemic.

**Results:**

The situation caused by restrictive quarantine measures related to the COVID-19 pandemic provokes aggressiveness and all forms of domestic violence.Clinical interview has revealed depression, anxiety, fear, low self-esteem, self-harm behavior, which formed the clinical picture of adaptation disorders, acute stress disorders, PTSR, psychosomatic disorders, substance abuse), sexual dysfunctions, eating disorders in the form of bulimia and anorexia.

**Conclusions:**

The study requires taking into account social, economic and gender characteristics. In all types of emergencies, including epidemics, violence against women tends to increase. This is facilitated by a number of factors, such as the crowding of the stay,a decrease in prosperity, provocation of alcohol abuse, state of uncertainty.

